# (*E*)-2-[2-(3-Fluoro­phen­yl)ethen­yl]quinolin-8-yl acetate

**DOI:** 10.1107/S1600536812030255

**Published:** 2012-07-10

**Authors:** Yan-Ping Huo, Xiao-Li Nie, Xiao-Ming Fang

**Affiliations:** a School of Chemical Engineering and Light Industry, Guangdong University of Technology, Guangzhou 510006, People’s Republic of China

## Abstract

In the crystal of the title compound, C_19_H_14_FNO_2_, the mol­ecules are linked by C—H⋯O hydrogen bonds in translational chains along the *b* axis. The dihedral angles formed by the quinoline system with the fluoro­benzene ring and the acet­oxy group are 8.15 (3) and 77.42 (4)°, respectively.

## Related literature

For the synthetic procedure, see: Zeng *et al.* (2006 ▶).
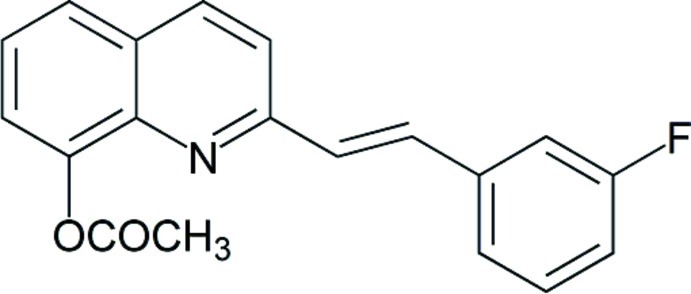


## Experimental

### 

#### Crystal data

C_19_H_14_FNO_2_
*M* *_r_* = 307.31
Monoclinic, 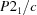

*a* = 17.628 (3) Å
*b* = 5.2641 (9) Å
*c* = 16.062 (3) Å
β = 100.528 (2)°
*V* = 1465.4 (4) Å^3^
*Z* = 4
Mo *K*α radiation
μ = 0.10 mm^−1^
*T* = 110 K
0.35 × 0.24 × 0.18 mm

#### Data collection

Bruker SMART CCD area-detector diffractometer
Absorption correction: multi-scan (*SADABS*; Sheldrick, 1996 ▶) *T*_min_ = 0.972, *T*_max_ = 0.982
8139 measured reflections
3177 independent reflections
2663 reflections with *I* > 2σ(*I*)
*R*_int_ = 0.017

#### Refinement

*R*[*F* ^2^ > 2σ(*F* ^2^)] = 0.044
*wR*(*F* ^2^) = 0.134
*S* = 1.02
3177 reflections
208 parameters
H-atom parameters constrained
Δρ_max_ = 0.67 e Å^−3^
Δρ_min_ = −0.26 e Å^−3^

### 

Data collection: *SMART* (Bruker, 2008 ▶); cell refinement: *SAINT* (Bruker, 2008 ▶); data reduction: *SAINT*; program(s) used to solve structure: *SHELXS97* (Sheldrick, 2008 ▶); program(s) used to refine structure: *SHELXL97* (Sheldrick, 2008 ▶); molecular graphics: *SHELXTL* (Sheldrick, 2008 ▶); software used to prepare material for publication: *SHELXTL*.

## Supplementary Material

Crystal structure: contains datablock(s) I, global. DOI: 10.1107/S1600536812030255/ld2060sup1.cif

Structure factors: contains datablock(s) I. DOI: 10.1107/S1600536812030255/ld2060Isup2.hkl

Supplementary material file. DOI: 10.1107/S1600536812030255/ld2060Isup3.cdx

Supplementary material file. DOI: 10.1107/S1600536812030255/ld2060Isup4.cml

Additional supplementary materials:  crystallographic information; 3D view; checkCIF report

## Figures and Tables

**Table 1:** Hydrogen-bond geometry (Å, °)

*D*—H⋯*A*	*D*—H	H⋯*A*	*D*⋯*A*	*D*—H⋯*A*
C1—H1*D*⋯O2^i^	0.98 (1)	2.54 (1)	3.495 (2)	166 (2)

Symmetry code: (i) 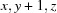.

**Fig. 1.**
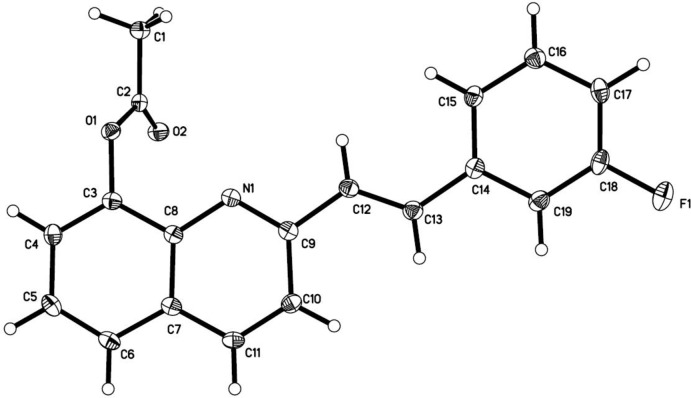
The asymmetric unit of the title compound.

## supplementary crystallographic information

### Comment

The (*E*)-2-[2-(3-fluorophenyl)ethenyl]-8-acetoxyquinoline was prepared *via* a reaction of 2-methyl-8-hydroxyquinaldine with 3-fluorobenzaldehyde according to Zeng *et al.* (2006). The molecular structure is shown on Fig. 1. There are non- classical intermolecular hydrogen bonds C–H···O between carbonyl oxygen and the methyl group (C···O = 3.495 (2) Å; C–H···O = 166°) connecting molecules into chains along the *b* axis.

### Experimental

The title compound was prepared by a method reported in the literature, see: Zeng *et al.* (2006). The crystals were obtained by dissolving the compound (0.1 g) in dichloromethane (5 ml) and then evaporating the solvent slowly at room temperature for about 3 days.

### Refinement

All H atoms were refined as riding atoms with isotropic displacement parameters 1.2 times larger or 1.5 times larger (methyl H) than the corresponding host carbon atoms. The methyl H atoms' positions were set based using AFIX 33 instruction in *SHELXL97* (Sheldrick, 2008)). The C—H distances were kept at 0.95 Å (0.98 Å for methyl hydrogens).

### Crystal data

**Table:**

C_19_H_14_FNO_2_	*F*(000) = 640
*M**_r_* = 307.31	*D*_x_ = 1.393 Mg m^−^^3^
Monoclinic, *P*2_1_/*c*	Mo *K*α radiation, λ = 0.71073 Å
Hall symbol: -P 2ybc	Cell parameters from 8139 reflections
*a* = 17.628 (3) Å	θ = 2.4–27.1°
*b* = 5.2641 (9) Å	µ = 0.10 mm^−^^1^
*c* = 16.062 (3) Å	*T* = 110 K
β = 100.528 (2)°	Plate, colorless
*V* = 1465.4 (4) Å^3^	0.35 × 0.24 × 0.18 mm
*Z* = 4	

### Data collection

**Table:**

Bruker SMART APEXII CCD area-detector diffractometer	3177 independent reflections
Radiation source: fine-focus sealed tube	2663 reflections with *I* > 2σ(*I*)
Graphite monochromator	*R*_int_ = 0.017
φ and ω scans	θ_max_ = 27.1°, θ_min_ = 2.4°
Absorption correction: multi-scan (*SADABS*; Sheldrick, 1996)	*h* = −22→22
*T*_min_ = 0.972, *T*_max_ = 0.982	*k* = −6→6
8139 measured reflections	*l* = −20→16

### Refinement

**Table:**

Refinement on *F*^2^	Primary atom site location: structure-invariant direct methods
Least-squares matrix: full	Secondary atom site location: difference Fourier map
*R*[*F*^2^ > 2σ(*F*^2^)] = 0.044	Hydrogen site location: inferred from neighbouring sites
*wR*(*F*^2^) = 0.134	H-atom parameters constrained
*S* = 1.02	*w* = 1/[σ^2^(*F*_o_^2^) + (0.0792*P*)^2^ + 0.6625*P*] where *P* = (*F*_o_^2^ + 2*F*_c_^2^)/3
3177 reflections	(Δ/σ)_max_ < 0.001
208 parameters	Δρ_max_ = 0.67 e Å^−^^3^
0 restraints	Δρ_min_ = −0.26 e Å^−^^3^

### Special details

**Table:**

Geometry. All e.s.d.'s (except the e.s.d. in the dihedral angle between two l.s. planes) are estimated using the full covariance matrix. The cell e.s.d.'s are taken into account individually in the estimation of e.s.d.'s in distances, angles and torsion angles; correlations between e.s.d.'s in cell parameters are only used when they are defined by crystal symmetry. An approximate (isotropic) treatment of cell e.s.d.'s is used for estimating e.s.d.'s involving l.s. planes.
Refinement. Refinement of *F*^2^ against ALL reflections. The weighted *R*-factor *wR* and goodness of fit *S* are based on *F*^2^, conventional *R*-factors *R* are based on *F*, with *F* set to zero for negative *F*^2^. The threshold expression of *F*^2^ > σ(*F*^2^) is used only for calculating *R*-factors(gt) *etc*. and is not relevant to the choice of reflections for refinement. *R*-factors based on *F*^2^ are statistically about twice as large as those based on *F*, and *R*- factors based on ALL data will be even larger.

### Fractional atomic coordinates and isotropic or equivalent isotropic displacement parameters (Å^2^)

**Table:**

	*x*	*y*	*z*	*U*_iso_*/*U*_eq_	
O1	0.34904 (6)	0.31492 (19)	0.74280 (6)	0.0242 (2)	
N1	0.26746 (7)	−0.0961 (2)	0.66528 (7)	0.0220 (3)	
F1	−0.13987 (6)	−0.95702 (19)	0.54984 (6)	0.0398 (3)	
O2	0.36690 (7)	0.0337 (2)	0.85047 (7)	0.0308 (3)	
C9	0.22819 (8)	−0.2876 (3)	0.62482 (8)	0.0225 (3)	
C8	0.34042 (8)	−0.0532 (3)	0.65088 (8)	0.0213 (3)	
C19	−0.02215 (9)	−0.7381 (3)	0.58416 (9)	0.0279 (3)	
H19A	0.0009	−0.8581	0.5523	0.033*	
C7	0.37520 (8)	−0.2014 (3)	0.59403 (8)	0.0225 (3)	
C14	0.02131 (8)	−0.5360 (3)	0.62403 (9)	0.0236 (3)	
C17	−0.13480 (8)	−0.5983 (3)	0.63768 (9)	0.0279 (3)	
H17A	−0.1874	−0.6201	0.6420	0.033*	
C3	0.38386 (8)	0.1525 (3)	0.69226 (8)	0.0224 (3)	
C12	0.14966 (8)	−0.3207 (3)	0.64139 (9)	0.0239 (3)	
H12A	0.1314	−0.1968	0.6760	0.029*	
C11	0.33106 (8)	−0.4031 (3)	0.55183 (8)	0.0248 (3)	
H11A	0.3517	−0.5070	0.5131	0.030*	
C4	0.45559 (8)	0.2107 (3)	0.67758 (9)	0.0254 (3)	
H4A	0.4828	0.3519	0.7053	0.031*	
C6	0.45038 (8)	−0.1410 (3)	0.58087 (9)	0.0263 (3)	
H6A	0.4739	−0.2420	0.5436	0.032*	
C5	0.48937 (8)	0.0617 (3)	0.62139 (9)	0.0277 (3)	
H5A	0.5395	0.1021	0.6116	0.033*	
C10	0.25884 (8)	−0.4474 (3)	0.56703 (9)	0.0248 (3)	
H10A	0.2289	−0.5835	0.5394	0.030*	
C16	−0.09130 (8)	−0.3979 (3)	0.67832 (9)	0.0279 (3)	
H16A	−0.1146	−0.2810	0.7110	0.033*	
C18	−0.09877 (9)	−0.7629 (3)	0.59126 (9)	0.0275 (3)	
C15	−0.01447 (8)	−0.3665 (3)	0.67175 (9)	0.0252 (3)	
H15A	0.0141	−0.2287	0.6999	0.030*	
C13	0.10159 (8)	−0.5092 (3)	0.61214 (9)	0.0262 (3)	
H13A	0.1211	−0.6386	0.5806	0.031*	
C2	0.34043 (8)	0.2298 (3)	0.82022 (9)	0.0241 (3)	
C1	0.29471 (9)	0.4184 (3)	0.86069 (11)	0.0334 (4)	
H1B	0.2887	0.3564	0.9166	0.050*	
H1C	0.2437	0.4403	0.8251	0.050*	
H1D	0.3218	0.5818	0.8668	0.050*	

### Atomic displacement parameters (Å^2^)

**Table:**

	*U*^11^	*U*^22^	*U*^33^	*U*^12^	*U*^13^	*U*^23^
O1	0.0275 (5)	0.0202 (5)	0.0253 (5)	0.0016 (4)	0.0058 (4)	−0.0001 (4)
N1	0.0229 (6)	0.0225 (6)	0.0209 (6)	0.0027 (4)	0.0049 (4)	0.0021 (4)
F1	0.0423 (6)	0.0331 (5)	0.0408 (6)	−0.0137 (4)	−0.0008 (4)	−0.0013 (4)
O2	0.0405 (6)	0.0254 (6)	0.0286 (5)	0.0033 (5)	0.0118 (5)	0.0030 (4)
C9	0.0236 (6)	0.0245 (7)	0.0190 (6)	0.0022 (5)	0.0028 (5)	0.0035 (5)
C8	0.0225 (6)	0.0221 (7)	0.0192 (6)	0.0030 (5)	0.0039 (5)	0.0043 (5)
C19	0.0344 (8)	0.0259 (7)	0.0238 (7)	−0.0037 (6)	0.0065 (6)	−0.0017 (6)
C7	0.0245 (7)	0.0250 (7)	0.0183 (6)	0.0044 (5)	0.0044 (5)	0.0046 (5)
C14	0.0273 (7)	0.0232 (7)	0.0198 (6)	−0.0022 (5)	0.0029 (5)	0.0034 (5)
C17	0.0227 (7)	0.0292 (8)	0.0302 (7)	−0.0035 (6)	0.0007 (6)	0.0087 (6)
C3	0.0254 (7)	0.0213 (7)	0.0207 (6)	0.0044 (5)	0.0050 (5)	0.0031 (5)
C12	0.0245 (7)	0.0261 (7)	0.0213 (6)	0.0015 (6)	0.0050 (5)	0.0006 (5)
C11	0.0297 (7)	0.0269 (7)	0.0184 (6)	0.0051 (6)	0.0061 (5)	−0.0002 (5)
C4	0.0250 (7)	0.0252 (7)	0.0253 (7)	−0.0018 (5)	0.0025 (5)	0.0044 (5)
C6	0.0266 (7)	0.0313 (8)	0.0227 (7)	0.0052 (6)	0.0089 (5)	0.0040 (6)
C5	0.0232 (7)	0.0348 (8)	0.0259 (7)	0.0002 (6)	0.0065 (6)	0.0070 (6)
C10	0.0275 (7)	0.0252 (7)	0.0208 (6)	0.0009 (6)	0.0017 (5)	−0.0011 (5)
C16	0.0265 (7)	0.0267 (8)	0.0301 (7)	0.0028 (6)	0.0042 (6)	0.0021 (6)
C18	0.0319 (7)	0.0233 (7)	0.0244 (7)	−0.0082 (6)	−0.0025 (6)	0.0044 (5)
C15	0.0256 (7)	0.0220 (7)	0.0266 (7)	−0.0016 (5)	0.0015 (6)	0.0011 (5)
C13	0.0288 (7)	0.0260 (7)	0.0245 (7)	−0.0007 (6)	0.0070 (6)	−0.0008 (5)
C2	0.0239 (6)	0.0222 (7)	0.0272 (7)	−0.0039 (5)	0.0070 (5)	−0.0016 (5)
C1	0.0351 (8)	0.0267 (8)	0.0424 (9)	0.0000 (6)	0.0177 (7)	−0.0047 (7)

### Geometric parameters (Å, º)

**Table:**

O1—C2	1.3561 (17)	C3—C4	1.3627 (19)
O1—C3	1.3962 (17)	C12—C13	1.334 (2)
N1—C9	1.3241 (19)	C12—H12A	0.9500
N1—C8	1.3667 (17)	C11—C10	1.360 (2)
F1—C18	1.3550 (17)	C11—H11A	0.9500
O2—C2	1.1986 (18)	C4—C5	1.407 (2)
C9—C10	1.430 (2)	C4—H4A	0.9500
C9—C12	1.4669 (19)	C6—C5	1.368 (2)
C8—C3	1.419 (2)	C6—H6A	0.9500
C8—C7	1.4221 (19)	C5—H5A	0.9500
C19—C18	1.382 (2)	C10—H10A	0.9500
C19—C14	1.396 (2)	C16—C15	1.387 (2)
C19—H19A	0.9500	C16—H16A	0.9500
C7—C11	1.414 (2)	C15—H15A	0.9500
C7—C6	1.4157 (19)	C13—H13A	0.9500
C14—C15	1.399 (2)	C2—C1	1.500 (2)
C14—C13	1.469 (2)	C1—H1B	0.9800
C17—C18	1.372 (2)	C1—H1C	0.9800
C17—C16	1.394 (2)	C1—H1D	0.9800
C17—H17A	0.9500		
			
C2—O1—C3	117.75 (11)	C5—C4—H4A	120.0
C9—N1—C8	117.80 (12)	C5—C6—C7	120.43 (13)
N1—C9—C10	122.74 (13)	C5—C6—H6A	119.8
N1—C9—C12	115.29 (12)	C7—C6—H6A	119.8
C10—C9—C12	121.95 (13)	C6—C5—C4	120.35 (13)
N1—C8—C3	119.39 (12)	C6—C5—H5A	119.8
N1—C8—C7	123.20 (13)	C4—C5—H5A	119.8
C3—C8—C7	117.39 (12)	C11—C10—C9	119.54 (13)
C18—C19—C14	119.78 (14)	C11—C10—H10A	120.2
C18—C19—H19A	120.1	C9—C10—H10A	120.2
C14—C19—H19A	120.1	C15—C16—C17	121.03 (14)
C11—C7—C6	123.02 (13)	C15—C16—H16A	119.5
C11—C7—C8	117.10 (12)	C17—C16—H16A	119.5
C6—C7—C8	119.87 (13)	F1—C18—C17	118.95 (14)
C19—C14—C15	118.19 (13)	F1—C18—C19	118.26 (14)
C19—C14—C13	118.33 (13)	C17—C18—C19	122.79 (14)
C15—C14—C13	123.46 (13)	C16—C15—C14	120.65 (14)
C18—C17—C16	117.54 (14)	C16—C15—H15A	119.7
C18—C17—H17A	121.2	C14—C15—H15A	119.7
C16—C17—H17A	121.2	C12—C13—C14	126.22 (14)
C4—C3—O1	118.91 (13)	C12—C13—H13A	116.9
C4—C3—C8	121.92 (13)	C14—C13—H13A	116.9
O1—C3—C8	118.86 (12)	O2—C2—O1	123.76 (13)
C13—C12—C9	125.75 (14)	O2—C2—C1	126.49 (14)
C13—C12—H12A	117.1	O1—C2—C1	109.74 (12)
C9—C12—H12A	117.1	C2—C1—H1B	109.5
C10—C11—C7	119.61 (13)	C2—C1—H1C	109.5
C10—C11—H11A	120.2	H1B—C1—H1C	109.5
C7—C11—H11A	120.2	C2—C1—H1D	109.5
C3—C4—C5	120.02 (14)	H1B—C1—H1D	109.5
C3—C4—H4A	120.0	H1C—C1—H1D	109.5

### Hydrogen-bond geometry (Å, º)

**Table:**

*D*—H···*A*	*D*—H	H···*A*	*D*···*A*	*D*—H···*A*
C1—H1*D*···O2^i^	0.98 (1)	2.54 (1)	3.495 (2)	166 (2)

Symmetry code: (i) *x*, *y*+1, *z*.
